# GLP-1 RA Improves Diabetic Retinopathy by Protecting the Blood-Retinal Barrier through GLP-1R-ROCK-p-MLC Signaling Pathway

**DOI:** 10.1155/2022/1861940

**Published:** 2022-11-03

**Authors:** Liufeng Wei, Weiwei Mo, Shanshan Lan, Haiyan Yang, Zhenxing Huang, Xinghuan Liang, Li Li, Jing Xian, Xuemei Xie, Yingfen Qin, Faquan Lin, Zuojie Luo

**Affiliations:** ^1^Department of Laboratory, The First Affiliated Hospital of Guangxi Medical University, No. 22 Shuangyong Road, Nanning, 530021 Guangxi, China; ^2^Department of Endocrinology, The First Affiliated Hospital of Guangxi Medical University, No. 6 Shuangyong Road, Nanning, 530021 Guangxi, China; ^3^Department of Renal Medicine, The Fourth Affiliated Hospital of Guangxi Medical University, No. 1 Liushi Road, Liuzhou, 545000 Guangxi, China

## Abstract

**Background:**

GLP-1 receptor agonists (GLP-1RA) are common clinical agents that are clinically protective against diabetic complications, such as diabetic retinopathy (DR). Previous studies have shown that the RhoA/ROCK pathway plays an important role in the development of DR. However, the specific mechanism of action between GLP-1RA and DR remains unclear. The aim of this study was thus to investigate the main mechanism involved in the protective effect of GLP-1RA on DR.

**Methods:**

Type 2 diabetic mice were fed a high-sugar, high-fat diet. Changes in the retinal structure were observed via HE staining and transmission electron microscopy. The expression of retinal GLP-1R, blood-retinal barrier- (BRB-) related proteins, inflammatory factors, and related pathway proteins were studied via Western blot or immunohistochemistry/immunofluorescence analysis.

**Results:**

GLP-1RA treatment reduced the blood glucose and lipid levels as well as the body weight of the diabetic mice while also improving retinal thickness, morphology, and vascular ultrastructure. Moreover, restored GLP-1R expression, increased Occludin and ZO-1 levels, and decreased albumin expression led to reduced retinal leakage and improved the BRB by inhibiting the RhoA/ROCK pathway.

**Conclusions:**

We found that the protective effect of GLP-1RA on the retina may be realized through the GLP-1R-ROCK-p-MLC signaling pathway.

## 1. Introduction

Diabetic retinopathy (DR) is one of the common complications of diabetes as well as one of the leading causes of blindness in the working population worldwide [1]. The blood-retinal barrier (BRB) is mainly divided into the outer retinal barrier (oBRB) and inner retinal barrier (iBRB); retinal vascular endothelial cells and pericytes, which share the same basement membrane, are the key cell components of the iBRB, and their structural and functional integrity is crucial for maintaining homeostasis in the retinal microenvironment. Tight junctions (TJ), adherens junctions, and gap junctions represent the molecular basis of iBRB integrity, and microvascular abnormalities resulting from the breakage or disruption of connexins include progressive thickening of the basement membrane, shedding/loss of endothelial and pericytes, and decellularized capillaries [2].

Rho GTPases belong to the Ras superfamily and are involved in cell migration, phagocytosis, contraction, and adhesion. ROCK (Rho-associated kinase) is considered to be one of the most important downstream targets of Rho and has been widely studied. Abnormal activation of ROCK has been confirmed in various vascular diseases, such as diabetic nephropathy, eye disease, heart disease, and hypertension. High glucose activates the RhoA/ROCK signaling pathway in blood vessels and induces the secretion of more tight junction proteins in vascular endothelial cells, which disrupts the equilibrium of mutual adhesion between vascular endothelial cells, resulting in endothelial cell dysfunction. Rho kinase inhibition has shown a beneficial role in the treatment of diabetic complications and can improve diabetic endothelial dysfunction, which is considered a promising target for the treatment of diabetic complications [3–5]. Since the RhoA/ROCK pathway is involved in regulating vascular contractility and intercellular adhesion as well as TJ by modulating endothelial cytoskeletal reorganization, the pharmacological inhibition of the RhoA/ROCK pathway may be beneficial in protecting the retina from diabetic damage.

Glucagon-like peptide 1 (GLP-1) is a small molecule peptide secreted by small intestinal L cells that stimulates insulin secretion in a glucose-dependent manner to exert hypoglycemic effects, and it is widely used clinically for the treatment of diabetes and its complications. An increasing number of studies have shown that GLP-1 ameliorates oxidative stress injury in the microvascular endothelial cells of mouse islets, attenuates islet *β*-cell dysfunction, [6] regulates retinal capillaries through GLP-1 receptor-PI3K/Akt-ENOS/NO-cGMP pathway and restores patency of retinal microvessels damaged by ischaemia-reperfusion, [7] inhibits the conversion of endothelial cells to mesenchymal cells after streptozotocin-induced intravascular injury in diabetic mice, attenuates the formation of denovo endothelium, [8] prevents lipopolysaccharide-induced neutrophil (PMN) extravasation, lung injury, and alveolar capillary barrier dysfunction, [9] protects mouse foot cells from high glucose-induced apoptosis by activating sirtuin 1 in vitro, and inhibits reactive oxygen species production and proinflammatory cytokine secretion [10]. In in vivo and in vitro models of myocardial ischemic injury, GLP-1 prevents cardiac insufficiency, reduces infarct size, and protects coronary vascular cells from oxidative stress injury, and its cardioprotective effects may be related to GLP-1R-mediated inactivation of the cAMP/PKA pathway and, subsequently, the RAGE/Rho/ROCK and MAPK signaling pathways [11, 12]. In conclusion, these findings suggest that GLP-1RA protect multiple organs from injury and prevent endothelial cell dysfunction. Therefore, in this study, we evaluated the effects of GLP-1RA on the retina of diabetic mice induced by high glucose and also explored the potential protective mechanisms.

## 2. Material and Methods

### 2.1. Animals

Thirty-six 5-week-old, 18-20 g male Balb/c mice were purchased from Slaughter Jingda Laboratory Animal Co., Ltd. (Hunan, China) and housed in the SPF-level laboratory of the Guangxi Medical University Laboratory Animal Center. Eight to ten mice were housed per cage in a standard temperature and humid environment with a 12-hour light/dark cycle and food and water provided ad libitum. After 16 hours of fasting, the mice were injected intraperitoneally with streptozotocin (45 mg/kg) dissolved in 0.1 M citrate buffer (pH 4.5) to induce hyperglycemia; control mice only received an equal amount of citrate buffer. Animals were considered diabetic when glucose levels ≥ 16.7 mmol/L for 3 consecutive days. Body weight changes were monitored weekly, and blood glucose levels were measured to confirm diabetic status. All experimental procedures were approved by the Animal Care & Welfare Committee of Guangxi Medical University.

### 2.2. Group Assignment and Drug Treatment

Thirty-six mice were randomly divided into the normal group (normal, *n* = 10) and modeling group (*n* = 26). The normal group was fed with general diet for 12 weeks, and the model group was fed with high-sugar and high-fat diet for 6 weeks + 5 days STZ + 6 weeks high-sugar and high-fat diet. After induction, the mice in the modeling group were randomly divided into the diabetic group (DM, *n* = 10), dulaglutide low-dose group (Du600 group, 600 *μ*g/kg/w, *n* = 8), and dulaglutide high-dose group (Du1000 group, 1000 *μ*g/kg/w, *n* = 8). The dulaglutide were administered subcutaneous weekly for 6 weeks. Mice in the normal and diabetic groups received saline daily under similar conditions.

### 2.3. Metabolic Testing

Tail vein blood was collected for glucose testing using a Roche blood glucose meter; in addition, the body weight of the mice was measured using an electronic balance. Total triglyceride (TG) and total cholesterol (TC) and high-density lipoprotein (HDL-C) and low-density lipoprotein (LDL-C) concentrations were measured using a fully automated biochemical analyzer.

### 2.4. Blood-Retinal Barrier Permeability

To analyze the extent of damage to the blood-retinal barrier in the Balb/c mice, the level of albumin leaking from the retinal vessels was assessed. After deep anesthesia, the thorax of the mice was opened for left ventricular perfusion. Subsequently, the mice were executed, and the retinas were isolated; their extraretinal vascular albumin levels were then assessed using the Western blot technique.

### 2.5. Hematoxylin and Eosin Staining

After fixation of retinal tissues, 4 micron sections were cut with a paraffin slicer. After dewaxing, the paraffin sections of retinal tissues were stained with hematoxylin-eosin staining solution at room temperature, cleaned and sealed with neutral resin, and the retinal thickness as well as morphological and structural changes was observed via light microscopy. Changes in retinal thickness were measured using Image-Pro Plus 6.0 for each group of three sections.

### 2.6. Immunohistochemistry Assay

After removal of the eye and fixation, the retinal tissue was sectioned with GLP-1R (Novus, Colorado, USA, Cat. No. NLS1205, 1 : 50), ROCK1, MMP-9, VEGF (Proteintech, Wuhan, China, Cat. No. 21850-1-AP, 1 : 2000), p-MLC (Bioss, Beijing, China, Cat. No. 10375-2-AP, 1 : 2000, Cat. No. 19003-1-AP, 1 : 2000), p-MLC (Bioss, Beijing, China, Cat. No. bs-4060R, 1 : 2000) Occludin (Signalway Antibody, Nanjing, China, Cat. No. 29275, 1 : 2000), and ZO-1 primary antibody (Biorbyt, Cambridge, UK, Cat. No. orb11587, 1 : 2000) were reacted overnight at 4°C, followed by goat anti-rabbit IgG (Signalway Antibody, Nanjing, China, Cat. No. L3012, 1 : 5000) secondary antibody for 60 min, visualized with DAB, and restained with hematoxylin and eosin (H&E). Finally, the films were sealed and photographed and observed with an Olympus microscope. The grayscale values of the positive colors of the above indicators for each group of three sections were analyzed using the ImageJ 18.0 mean positive staining area percentage method, which is the %Area measured.

### 2.7. Immunofluorescence Assay

The primary antibody was operated as in 2.6 immunohistochemistry before incubation, followed by incubation with Alexa Fluor 488 (Proteintech, Wuhan, China, Cat. No. SA00013-1, 1 : 1000) as secondary antibody for 60 min. Fluorescence was visualized by DAPI and blocked with antiquenching agent. After blocking, the slices were photographed and observed with an Olympus microscope.

### 2.8. Transmission Electron Microscopy

The intact mouse eyeballs were fixed in electron microscopy fixative at 4°C for 2-4 h followed by 1% osmium acid for 2 h, dehydrated in ethanol step-by-step, cured via immersion in an embedding solution, ultrathin sections after semithin positioning, and observed under a transmission electron microscope after double staining with uranium lead.

### 2.9. Western Blotting Assay

After retinal detachment, total retinal protein extracts were obtained by tissue lysate. Subsequently, the protein concentration was determined using the BCA Protein Analysis Kit. The proteins were separated by SDS/PAGE and transferred to a polyvinylidene fluoride membrane, incubated with 5% skim milk powder, and sealed. The protein-containing membrane was incubated with anti-GLP-1R (Novus, Colorado, USA, Cat. No. NLS1205, 1 : 500), anti-Albumin (Proteintech, Wuhan, China, Cat. No. 16475-AP, 1 : 5000), and anti-*β*-tubulin (Abmart, Shanghai, China, Cat. No. M30109S, 1 : 2000) antibodies at 4°C overnight. Then, the membrane and horseradish peroxidase-bound secondary antibody (Signalway Antibody, Cat. No. L3012) were placed at 37°C for 1 h. The LI-COR system was then used to visualize and confirm the protein. ImageJ 18.0 was used to analyze the intensity of the bands of interest.

### 2.10. Statistical Analysis

SPSS 22.0 was used for statistical analysis, expressed as the means ± SEM. One-way ANOVA was followed by Bonferroni's post hoc test. Prism 5 software (GraphPad) was used for all statistical mapping, with differences considered statistically significant at *p* < 0.05.

## 3. Results

### 3.1. GLP-1RA Reduce Blood Glucose and Lipid Levels in Diabetic Mice While Also Reducing Body Weight

In this study, 36 mice were randomly divided into four groups; their body weight was measured at the beginning of the first week; blood glucose levels were measured at the beginning of the fourth week, and lipid levels were measured via heart blood collection at the end of the experiment at the 12th week. The body weight of the mice in the DM group was significantly lower compared to those in the normal group (*p* < 0.05), while the body weight of the mice in the GLP-1RA group was maintained at a relatively stable level throughout the entire study period (Table 1(a)). In terms of the blood glucose levels of the modeling group, by week 7, there was no change in diabetic status compared to the first induced diabetes—that is, all diabetic animals maintained their diabetic status. The blood glucose levels were significantly higher in the DM group when compared to the normal group (*p* < 0.05); compared to the DM group, the blood glucose was reduced by 44.8% at 8 weeks, 67.4% at 10 weeks, and 61.2% at 12 weeks in the Du1000 group (Table 1(b)). In terms of lipid levels, the TG levels were much higher in the DM group than in the normal group at 12 weeks, while the TG levels were lower after GLP-1RA treatment; TC, HDL, and LDL showed similar trends (Table 1(c)). These results suggest that GLP-1RA can lower blood glucose levels and lipid levels while reducing body weight in diabetic mice.

### 3.2. GLP-1R Expression in the Retina

**Figure 1:**
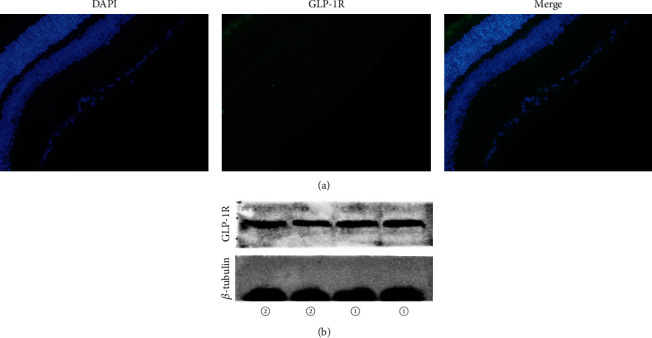
Expression of GLP-1R. (a) Immunofluorescence: GLP-1R was expressed in various layers of mouse retina (40x). (b) Western blot: GLP-1R was expressed in the mouse retina and INS 1 cells (rat insulinoma cells were used as positive control: (1) rat insulinoma cells and (2) mouse retinal tissue).

Green fluorescence revealed the expression of GLP-1R in the mouse retina, as shown in Figure 1(a); specifically, GLP-1R was expressed in the inner layers (i.e., the GCL layer, IPL layer, and INL layer). In addition, the Western blot results also showed that GLP-1R was expressed in the retina (Figure 1(b)).

### 3.3. GLP-1RA Improve Retinal Morphological Changes in Diabetic Mice

**Figure 2:**
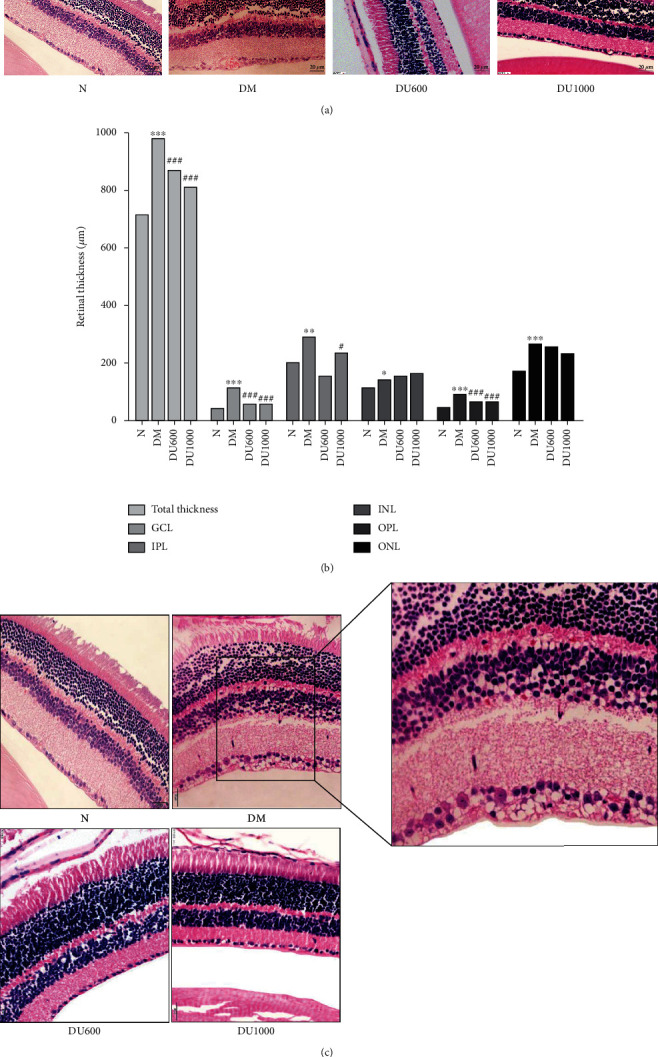
Overall retinal thickness and thickness of each layer and morphological changes. (a) Overall retinal thickness and thickness of each layer (40x). (b) Histogram comparison of overall retinal thickness and thickness of each layer. (c) Morphological changes of the retina (40x). Annotation: compared with the normal group, ^∗^*p* < 0.05, ^∗∗^*p* < 0.01, and ^∗∗∗^*p* < 0.001; compared with the DM group, ^#^*p* < 0.05, ^##^*p* < 0.01, and ^###^*p* < 0.001.

In the HE staining results, as shown in Figure 2, the total retinal thickness and the thickness of each layer (IPL, INL, OPL, and ONL layers) were significantly increased in the DM group at 12 weeks when compared with the normal group (*p* < 0.05) (Figures 2(a) and 2(b)). The total retinal thickness and the thickness of each layer were reduced to varying degrees after GLP-1RA treatment. In the normal group, the structure of the ten layers of the retina was clearly visible, neatly arranged, and without retinal capillary dilatation. Compared with the normal group, the total retinal thickness in the DM group was significantly increased and disordered, with vascular dilatation in the retinal tissue layer and vacuolar degeneration in the cells of the GCL and INL layers. After treatment with the GLP-1RA, the total thickness of the retina was significantly reduced; the alignment of the layers was neater than in the DM group; the vacuolar degeneration of the cells in the GCL and INL layers improved; the symptom of dilated capillaries in the tissue layer improved (Figure 2(c)). Therefore, it can be suggested that GLP-1RA may play a protective role in retinal morphology in diabetic rats.

### 3.4. Improvement of Retinal Ultrastructure in Diabetic Mice by GLP-1RA

**Figure 3:**
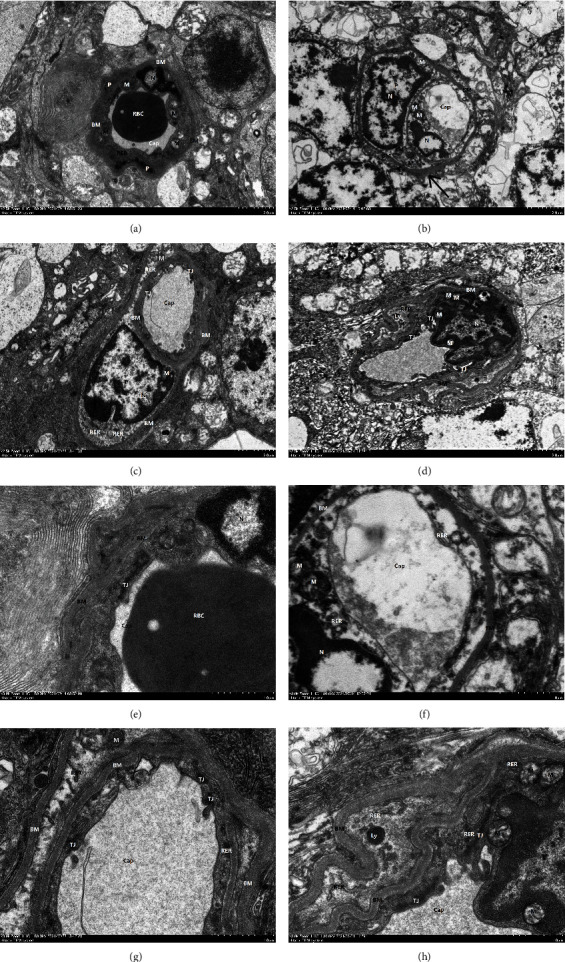
Ultrastructural changes in the retina of mice in each group. (a–d) Normal group, DM group, DU600 group, and DU1000 group (×2500). (e–h) The corresponding high magnification field of view for groups (a–d) (×8000).

We used transmission electron microscopy to observe the improvement effect of GLP-1RA on the retinal ultrastructure of diabetic mice, as shown in Figures 3(a)–3(h). The vascular structure of the normal group was clear; the lumen was not obviously collapsed; erythrocytes were visible in the lumen; the morphological structure of the endothelial cells was not significantly abnormal; there was no obvious edema; there was TJ between the endothelial cells; the cell junction gap was fair and tightly connected; the basement membrane was clear and uniform in thickness, and no obvious edema was seen in the pericytes (Figures 3(a) and 3(e)). Compared with the normal group, the DM group exhibited obvious edema in the blood vessels and their surrounding neural tissue structures; the lumen of the blood vessels was compressed; flocculent protein aggregates were seen in the lumen; the endothelial cells exhibited obvious edema and a shallow cytoplasmic density; the structure of the TJ between the endothelial cells was destroyed; the dense areas were discontinuous and loosely connected; the basement membrane was thickened; a small number of local areas were obviously thickened (black arrows); the pericytes exhibited obvious edema, and the mitochondria were individually vacuolated (Figures 3(b) and 3(f)). After treatment with the GLP-1RA, the vascular structure was clearer than that of the DM group; the lumen did not show obvious collapse; the degree of edema in the endothelial cells was reduced; the structure of the TJ between the endothelial cells was tighter than before; the thickening of the basement membrane was improved, and the degree of edema in the pericytes was reduced; the degree of improvement in terms of all of the above was better in the high-dose group than in the low-dose group (Figures 3(c), 3(d), 3(g), and 3(h)).

### 3.5. Protective Effect of GLP-1RA on the Blood-Retinal Barrier in Diabetic Mice

**Figure 4:**
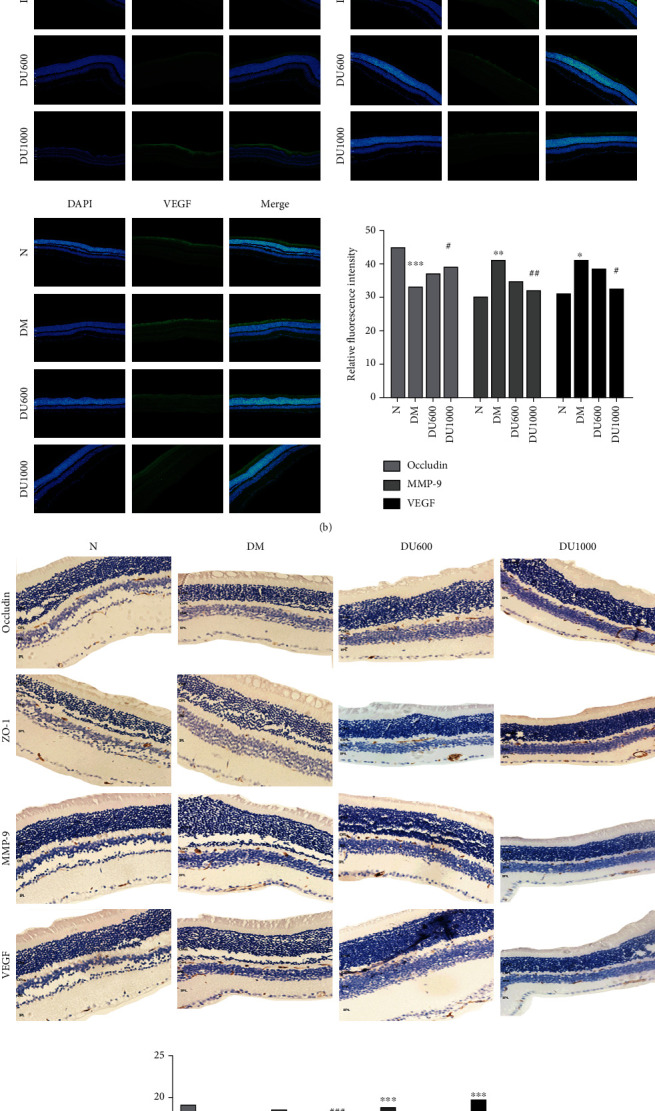
Reduction of albumin leakage and MMP-9/VEGF expression and increasing of ZO-1/Occludin expression through GLP-1RA treatment. (a) Western blot analysis of retinal albumin leakage in four groups. (b, c) Immunofluorescence and immunohistochemistry analysis of retinal MMP-9/VEGF/ZO-1/Occludin expression in four groups and histogram comparisons (40×). Annotation: compared with the normal group, ^∗^*p* < 0.05, ^∗∗^*p* < 0.01, and ^∗∗∗^*p* < 0.001; compared with the DM group, ^#^*p* < 0.05, ^##^*p* < 0.01, and ^###^*p* < 0.001.

To assess the damage of the BRB in the mice, we examined the level of albumin leakage from the retinal vessels. As shown in (Figure 4(a)), the albumin leakage was significantly increased in the DM group and significantly decreased following GLP-1RA treatment, with the leakage decreasing with higher doses.

Then, we detected the expression of retinal TJ proteins Occludin and ZO-1 as well as inflammatory factors MMP-9 and VEGF via immunofluorescence and immunohistochemistry, and the results showed that, compared with the normal group, the expression of Occludin and ZO-1 was significantly decreased, and the expression of MMP-9 and VEGF was significantly increased in the DM group at 12 weeks; moreover, the above changes could be reversed by GLP-1RA treatment (Figures 4(b) and 4(c)). Therefore, GLP-1RA treatment could increase the expression of TJ proteins Occludin and ZO-1 to protect against BRB disruption by reducing the albumin leakage and inflammatory factor expression.

### 3.6. Effect of GLP-1RA on RhoA/ROCK1 Pathway in Diabetic Mice

**Figure 5:**
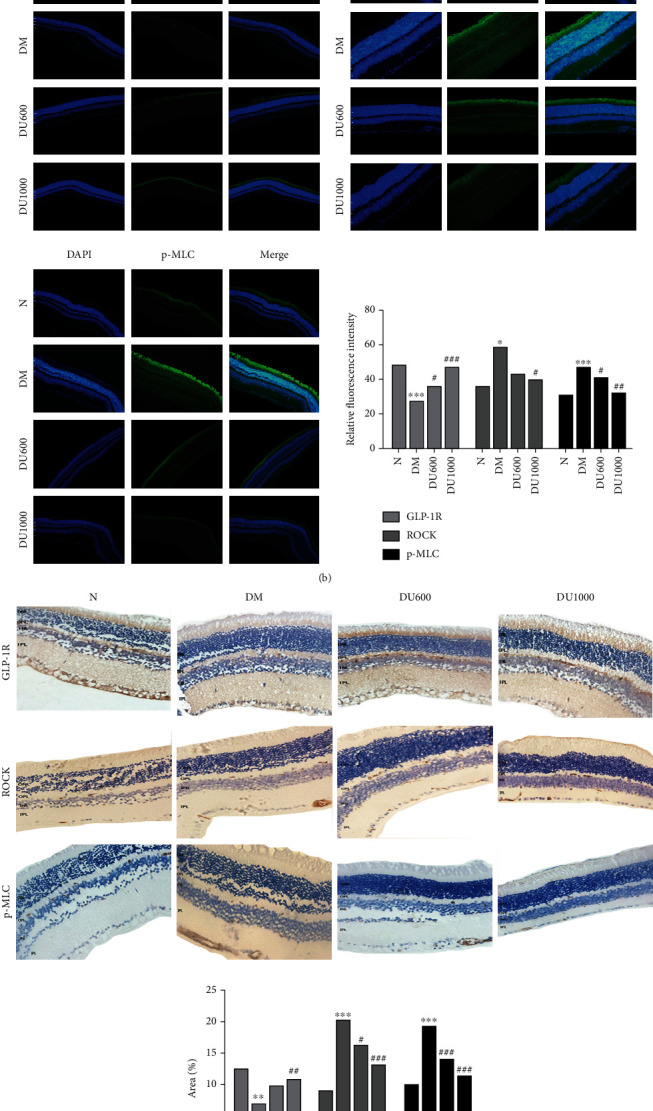
Reduced ROCK/p-MLC expression and increased GLP-1R expression through GLP-1RA treatment. (a) Western blot analysis of retinal GLP-1R protein in four groups.(b, c) Immunofluorescence and immunohistochemistry analysis of retinal GLP-1R ROCK/P-MLC expression in four groups and histogram comparisons (40×). Annotation: compared with the normal group, ^∗^*p* < 0.05, ^∗∗^*p* < 0.01, and ^∗∗∗^*p* < 0.001; compared with the DM group, ^#^*p* < 0.05, ^##^*p* < 0.01, and ^###^*p* < 0.001.

GLP-1RA acts mainly by binding to GLP-1 receptor (GLP-1R) through the retina; so, we used Western blot, immunofluorescence, and immunohistochemistry to detect the expression of GLP-1R and related pathway proteins. Our results showed that, at 12 weeks, GLP-1R expression decreased and ROCK1 and p-MLC expression increased significantly. However, GLP-1RA treatment restored the GLP-1R expression, while decreasing ROCK1 and p-MLC expressions when compared with the DM group; also, the improvement in the high-dose group was greater than that in the low-dose group (Figures 5(a)–5(c)).

## 4. Discussion

DR is one of the serious complications of diabetes—often leading to vision loss, and, in severe cases, retinal detachment and even blindness. The global prevalence of DR among diabetes patients is 22.27%, and the number of people with DR worldwide was estimated to be 103.12 million in 2020; by 2045, this number is expected to increase to 160.5 million [13].

The main features of diabetes are hyperglycemia and systemic chronic low-grade inflammation, which can lead to endothelial dysfunction, and endothelial damage is the initial process in the progression of diabetic vascular complications [14, 15]. Traditionally, DR has been thought of as a microvascular complication associated with endothelial dysfunction, with the early and most significant change being a disruption in the BRB. However, DR is increasingly being considered a neurovascular disease rather than just a microvascular lesion [16, 17]. The BRB is a barrier between the blood and atrial fluid, lens, and vitreous humor of the eye and plays an important role in maintaining normal retinal function. The iBRB is composed of retinal vascular endothelial cells as well as their connections, neurons, the basement membrane, pericytes, and other structures [2].

Numerous studies have shown that the most important pathological basis and morphological changes in the early stages of DR occur in the iBRB, which is directly damaged by exposure to a hyperglycemic environment [18, 19]. In our study, the BRB was disrupted in the DR mice, which was mainly manifested by extensive vascular edema and luminal compression, the endothelial cells being significantly edematous, the impairment of the intercellular TJ, the thickening of the basement membrane, and the pericytes being edematous. In the course of DR lesions, the disruption of the BRB causes retinal hemorrhage and exudation, leading to the development of retinal lesions. Our study showed that retinal albumin leakage and inflammatory factors were significantly increased, and TJ protein expression was decreased in the DR mice, while this change was significantly reversed by GLP-1RA treatment. Thus, here, we demonstrated that GLP-1RA can effectively inhibit albumin leakage in the retina of diabetic mice by repairing TJ.

The pharmacological modulation of GLP-1 has emerged as a new therapeutic option for DM, but its short half-life and degradation by DPP-4 upon entry into circulation prevent its full effect. GLP-1RA are enterostatin analog, with hypoglycemic, hypotensive, and weight-reducing effects; additionally, they also have long-term cardiovascular protective effects [20]. In clinical studies, it has been shown that the hypoglycemic and weight loss efficacy of GLP-1RA is maintained by long-term use [21–25]. A recently published study reported significant weight loss in patients at 36 weeks with weekly dulaglutide use, with higher doses resulting in greater weight loss [21]. In contrast, our study showed that GLP-1RA treatment significantly reduced the blood glucose levels and lipid levels in the DR mice but did not have a significant weight loss effect, probably because our study period was not long enough to observe any such weight loss effect of GLP-1 on the mice.

Due to the protective effect of GLP-1 on the retina, are all the effects observed in the GLP-1-treated group direct effects of GLP-1 on the retina, or are they related to the ability of GLP-1 to increase systemic insulin secretion or lower blood glucose levels? In one study, after three weeks of treatment in db/db mice with GLP-1 eye drops or vectors, the topical administration of GLP-1 was found to reverse reactive gliosis and albumin extravasation and prevent apoptosis and retinal dysfunction. The topical administration of GLP-1 using eye drops was also reported in a previous study by the same authors as preventing retinal neurodegeneration and early vascular leakage in 12-week-old db/db mice [26, 27]. A recent study also showed that three different routes of administration (subcutaneous injection, intravitreal injection, and eye drops) did not result in systemic glycemic alterations, which demonstrated that the exendin-4 vasoprotective effects were not associated with lowering blood glucose levels or altering insulin levels [7]. GLP-1R activation was also found to ameliorate cognitive decline in Type 2 diabetes through metabolism-independent pathways [28]. It is worth noting that the topical use of GLP-1 eye drops could not significantly pass through the systemic circulation to lower the blood glucose levels; therefore, the observed effect of GLP-1 was attributed to a direct local effect unrelated to the improved metabolic control. In contrast, in our study, we were unable to observe the effect of the systemic use of GLP in terms of retinal improvement independent of its glucose-lowering effect. Other important reasons for the use of topical GLP-1 administration in the clinical setting include its potential for self-administration and its limited effect in the eye, thus minimizing the associated systemic effects.

The small G protein family is widely distributed in mammalian tissue cells and exhibits GTPase activity, also known as Rho guanosine triphosphatases (RhoGTPases), which are major regulators of the cytoskeleton. The RhoA/ROCK signaling pathway has been found to be associated with various physiological functions, such as vascular and tissue permeability, tissue contraction, and growth [3, 4]. It is characterized by the reorganization of the actin cytoskeleton by contracting actin myosin, which culminates in the formation of intercellular gaps. Moreover, the phosphorylation of its downstream substrate myosin light chain (MLC) induces actin contraction and the disruption of endothelial cell adhesion [29, 30]. ROCK inhibition appears to be a promising therapeutic concept targeting various pathogenic mechanisms in the diabetic retina. In vitro, ROCK1 is activated by high glucose levels in human retinal capillary endothelial cells, which induces their proliferation. [5] Meanwhile, in human and mouse models, ROCK-1 activation and reduced levels of Occludin, ZO-1, and claudin-5 proteins under hyperglycemic conditions disrupt the TJ of endothelial cells, leading to iBRB disruption, while ROCK inhibition restores endothelial claudin-5 expression on cell membranes, maintains ZO-1 expression, and restores vascular barrier dysfunction [31]. Our study showed that, in a hyperglycemic state, the RhoA/ROCK signaling pathway in the retina activates and leads to phosphorylation of the downstream substrate MLC, which increases retinal vascular permeability and leads to iBRB breakdown by reducing/disrupting TJ. Our data further indicated that GLP-1 prevents a high glucose-induced increase in retinal vascular permeability and vascular leakage in mice by binding to GLP-1R on the retina and inhibiting the activation of the RhoA/ROCK pathway.

We have shown that exposure to high glucose levels promotes the activity of the RhoA/ROCK signaling pathway in the retina of DR mice—with both ROCK and p-MLC upregulated; meanwhile, GLP-1 treatment downregulates both proteins. Therefore, the potential protective effect of GLP-1 on the iBRB in DR mice may be through the inhibition of ROCK and p-MLC, a reduction in the retinal vascular permeability, and the upregulation of TJ, and this protective effect is exerted through GLP-1R.

## Figures and Tables

**Table 1a:** (a) Body weight changes of mice

Group	*n*	Body weight (g)				
		4 w	6 w	8 w	10 w	12 w
Normal	10	27.86 ± 2.67	29.59 ± 2.48	30.06 ± 3.05	31.22 ± 2.93	30.50 ± 2.56
DM	10	25.42 ± 2.05	22.73 ± 1.68^∗∗∗^	23.81 ± 2.59^∗∗∗^	24.20 ± 2.59^∗∗∗^	24.20 ± 2.52^∗∗∗^
DU600	8	26.51 ± 1.59	24.82 ± 1.39^#^	22.58 ± 1.44	23.76 ± 1.88	23.83 ± 2.35
DU1000	8	26.17 ± 1.75	23.51 ± 1.54	23.13 ± 1.74	22.86 ± 1.39	25.63 ± 2.55

**Table 1b:** (b) Blood glucose changes of mice

Group	*n*	Blood glucose (mmol/L)				
		4 w	6 w	8 w	10 w	12 w
Normal	10	6.08 ± 0.91	7.19 ± 0.97	6.85 ± 1.67	6.96 ± 0.92	5.12 ± 0.75
DM	10	20.75 ± 3.85^∗∗∗^	22.54 ± 3.81^∗∗∗^	27.45 ± 4.02^∗∗∗^	28.47 ± 4.74^∗∗∗^	25.99 ± 4.07^∗∗∗^
DU600	8	19.75 ± 1.00	24.02 ± 0.82	15.13 ± 5.72^###^	13.27 ± 5.19^###^	10.41 ± 4.18^###^
DU1000	8	25.35 ± 3.07	22.92 ± 2.32	15.13 ± 3.80^###^	9.26 ± 2.98^###^	10.07 ± 2.21^###^

**Table 1c:** (c) Blood lipid index changes of mice

Group	*n*	Serum index (mmol/L)			
		TC	TG	LDL	HDL
Normal	10	3.64 ± 0.32	0.72 ± 0.25	0.44 ± 0.16	1.91 ± 0.20
DM	10	4.57 ± 0.74^∗∗^	1.15 ± 0.35^∗∗^	0.59 ± 0.44	2.39 ± 0.46^∗∗^
DU600	8	4.02 ± 0.62	1.00 ± 0.42	0.45 ± 0.21	2.26 ± 0.30
DU1000	8	3.94 ± 0.72^#^	0.82 ± 0.28^#^	0.38 ± 0.08	2.12 ± 0.36

Annotation: compared with the normal group, ^∗^*p* < 0.05, ^∗∗^*p* < 0.01, and ^∗∗∗^*p* < 0.001; compared with the DM group, ^#^*p* < 0.05, ^##^*p* < 0.01, and ^###^*p* < 0.001.

## Data Availability

My manuscript already included a data availability statement.

## Supplementary Materials

Supplementary Materials The results of transmission electron microscopy, with the measurement scale.
