# Supplementary material for: GLP-1 RA Improves Diabetic Retinopathy by Protecting the Blood-Retinal Barrier through GLP-1R-ROCK-p-MLC Signaling Pathway
Source: J Diabetes Res. 2022 Nov 3;2022:1861940. doi: 10.1155/2022/1861940 (PMC9649324; doi:10.1155/2022/1861940)
Supplement: Supplementary Materials — The results of transmission electron microscopy, with the measurement scale. [file 1861940.f1.pdf]

Figure 3 shows the results of transmission electron microscopy, with the measurement scale under the figure.

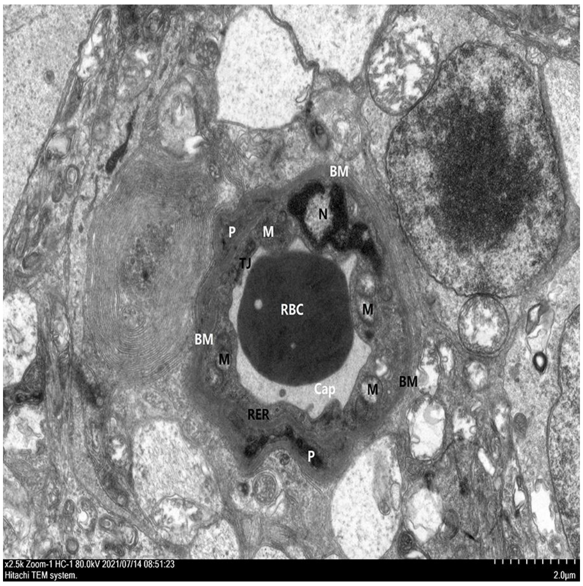

N 2500x

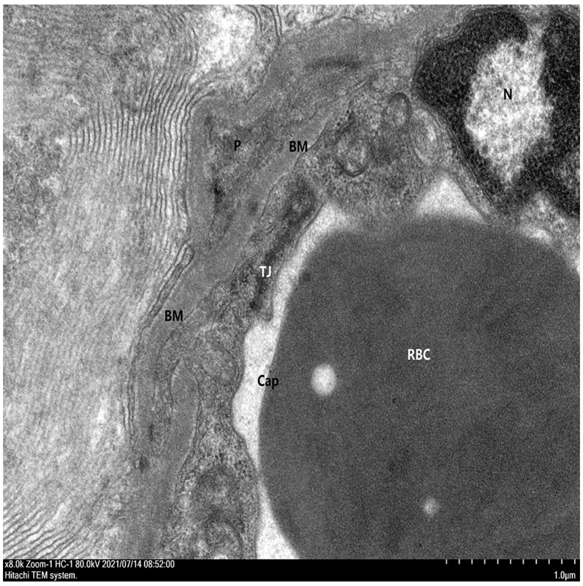

N 8000x

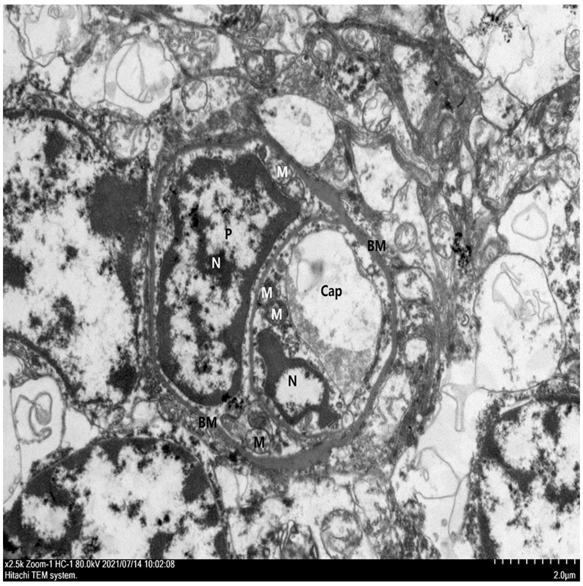

HG 2500x

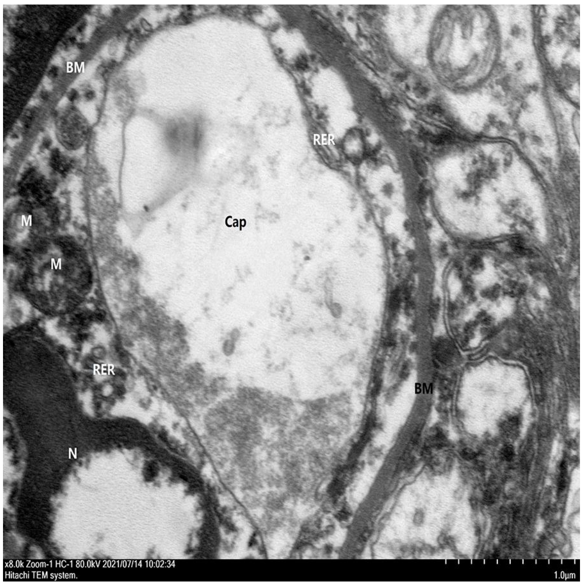

HG 8000x

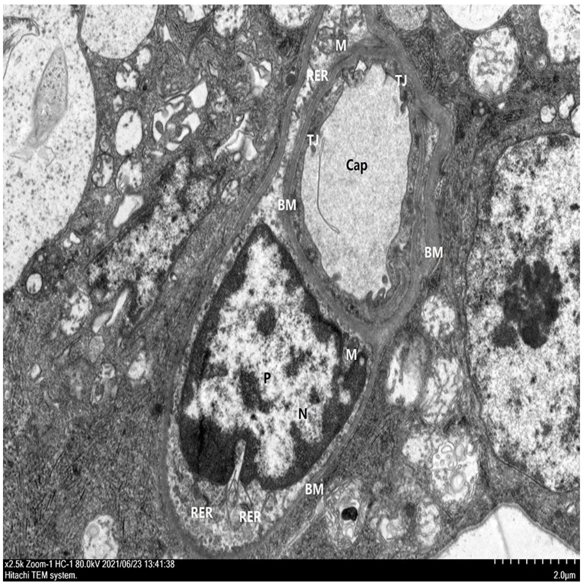

600 2500x

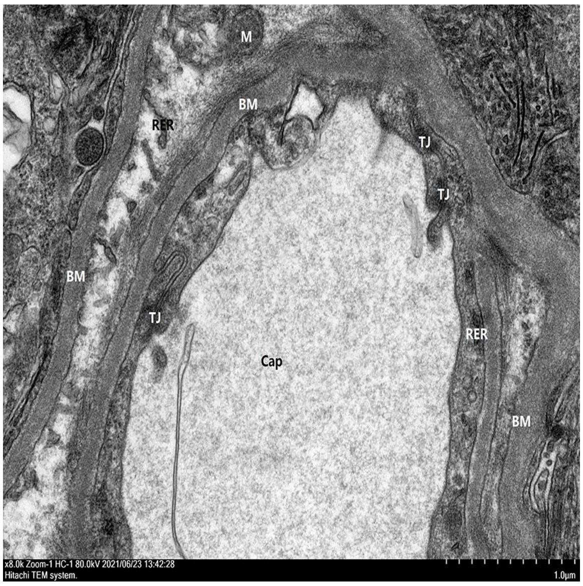

600 8000x

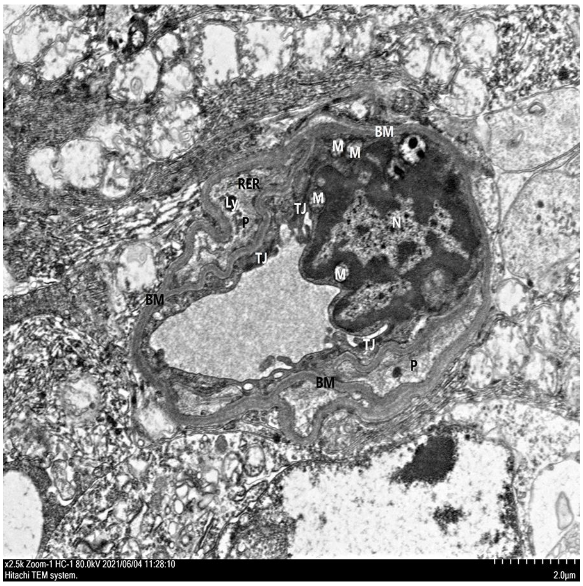

1000 2500x

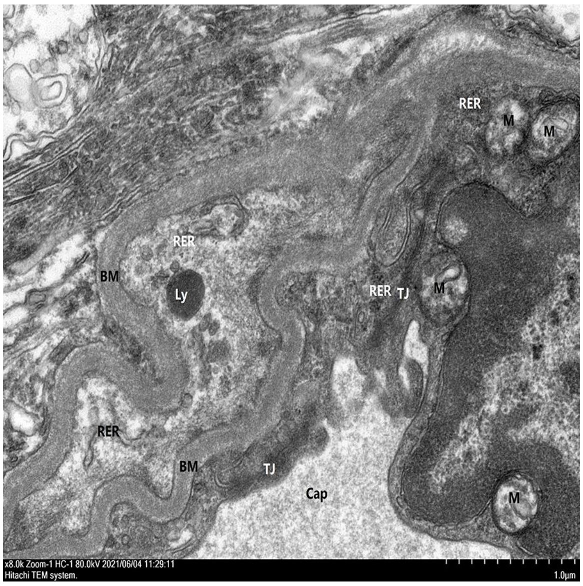

1000 8000x
